# Metastasis of Tumor Cells Is Enhanced by Downregulation of Bit1

**DOI:** 10.1371/journal.pone.0023840

**Published:** 2011-08-23

**Authors:** Priya Prakash Karmali, Chris Brunquell, Hau Tram, Shubha Kale Ireland, Erkki Ruoslahti, Hector Biliran

**Affiliations:** 1 Sanford-Burnham Medical Research Institute, La Jolla, California, United States of America; 2 Sanford-Burnham Medical Research Institute, Santa Barbara, California, United States of America; 3 Department of Biology, Xavier University of Louisiana, New Orleans, Louisiana, United States of America; Technische Universität München, Germany

## Abstract

**Background:**

Resistance to anoikis, which is defined as apoptosis induced by loss of integrin-mediated cell attachment to the extracellular matrix, is a determinant of tumor progression and metastasis. We have previously identified the mitochondrial Bit1 (Bcl-2 inhibitor of transcription) protein as a novel anoikis effector whose apoptotic function is independent from caspases and is uniquely controlled by integrins. In this report, we examined the possibility that Bit1 is suppressed during tumor progression and that Bit1 downregulation may play a role in tumor metastasis.

**Methodology/Principal Findings:**

Using a human breast tumor tissue array, we found that Bit1 expression is suppressed in a significant fraction of advanced stages of breast cancer. Targeted disruption of Bit1 via shRNA technology in lowly aggressive MCF7 cells conferred enhanced anoikis resistance, adhesive and migratory potential, which correlated with an increase in active Extracellular kinase regulated (Erk) levels and a decrease in Erk-directed phosphatase activity. These pro-metastasis phenotypes were also observed following downregulation of endogenous Bit1 in Hela and B16F1 cancer cell lines. The enhanced migratory and adhesive potential of Bit1 knockdown cells is in part dependent on their high level of Erk activation since down-regulating Erk in these cells attenuated their enhanced motility and adhesive properties. The Bit1 knockdown pools also showed a statistically highly significant increase in experimental lung metastasis, with no differences in tumor growth relative to control clones *in vivo* using a BALB/c nude mouse model system. Importantly, the pulmonary metastases of Bit1 knockdown cells exhibited increased phospho-Erk staining.

**Conclusions/Significance:**

These findings indicate that downregulation of Bit1 conferred cancer cells with enhanced anoikis resistance, adhesive and migratory properties *in vitro* and specifically potentiated tumor metastasis *in vivo*. These results underscore the therapeutic importance of restoring Bit1 expression in cancer cells to circumvent metastasis at least in part through inhibition of the Erk pathway.

## Introduction

The loss of the anchorage dependence of normal epithelial cells is one of the hallmarks of malignant cells [1], [2] and is acquired through resistance to detachment-induced apoptosis (anoikis) [3]. The ability of tumor cells to survive detachment and evade anoikis may enable them to leave their original site, invade the surrounding tissue and extracellular matrix, enter the blood or lymphatic circulation, and eventually metastasize to secondary sites. Since the acquisition of anoikis resistance is an important determinant of transformation and metastatic potential [4], suppression of the anoikis pathway may contribute to tumorigenic and metastatic proficiency of malignant cells. This work analyzes the potential role of the novel anoikis effector Bit1 in tumor progression and metastasis.

Bit1 (Bcl2-inhibitor of transcription) is a mitochondrial protein that is released to the cytosol following loss of cell attachment and interacts with the Groucho/TLE family transcription factor AES to induce caspase-independent apoptosis. Importantly, the Bit1 apoptotic activity can only be counteracted by intergrin-mediated cell attachment and not by any anti-apoptotic factor such as bcl2, bcl-xl and Akt [5]. Thus, Bit1 is sometimes regarded as a guardian of anchorage-dependence. The anoikis function of Bit1 has been demonstrated in several transformed and tumor cell lines. While overexpression of mitochondrial Bit1 in cells enhances their sensitivity to anoikis, downregulation of Bit1 expression renders tumor cells as well as normal cells more resistant to anoikis [6]. Despite the well documented data on the potent pro-anoikis effect of Bit1 on tumor cells, its role in tumor progression and metastasis remains unknown.

There is evidence to suggest that the Bit1 anoikis pathway is important in tumorigenesis. First, we have recently shown that Bit1 negatively regulates the extracellular signal-regulated kinase 1/2 (Erk 1/2) survival signaling, a pathway which is activated in several malignacies [7], [8], [9]. Mouse embryonic fibroblasts (MEFs) from Bit1 knockout mice and tumor cells in which Bit1 had been downregulated by siRNA interference showed a marked increase in Erk activation [10], and such elevated Erk activity in part accounts for the enhanced anoikis resistance in Bit1 knockdown cells. Considering the crucial role of the Erk signaling pathway in tumorigenesis and metastasis [7], [8], [9], the Bit1-mediated Erk regulation may influence tumorigenesis and/or the metastatic process. Second, TLE1, a groucho transcriptional regulator that counteracts Bit1/AES apoptosis, is an indicator of poor prognosis in lymphoma patients [11] and a putative lung-specific oncogene [12]. Recently, AES has been shown to be a suppressor of colon cancer metastasis [13]. Taken together, these findings implicate the Bit1/AES pathway in tumorigenesis and/or metastasis.

Considering the anoikis remains a critical barrier to transformation and metastasis, we examined the possibility that suppression or nonfunctionality of the Bit1 anoikis pathway may contribute to tumor progression. In the present study, we found that Bit1 expression is significantly downregulated in advanced cases of breast cancer tissues as compared to the counterpart normal breast tissue. Importantly, stable knockdown of Bit1 expression in lowly aggressive breast cancer MCF7 as well as in B16F1 and Hela cells resulted in enhanced anoikis resistance, adhesion, and migratory property. Consistent with these *in vitro* data, the stable Bit1 knockdown cells also showed enhanced metastasis *in vivo*. These findings implicate a role of Bit1 in metastasis.

## Results

### Expression of Bit1 is reduced in invasive breast tumors

**Figure 1:**
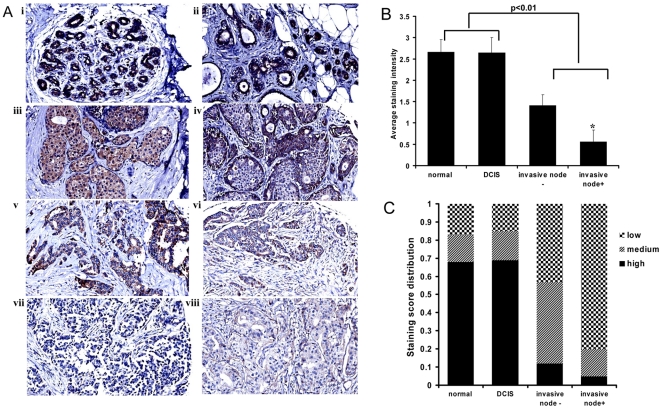
Bit1 expression is downregulated in invasive breast tumors. A. Breast tumor tissue array slides were stained with affinity purified anti-Bit1 (Sigma). Images are representative of each respective case type: normal breast 10X (i,ii), Ductal carcinoma in situ (DCIS) 10X (iii, iv), node negative invasive breast carcinoma 10X (v, vi), and node positive invasive breast carcinoma 10x (vii, viii). B. The average staining intensity of each subgroup was determined. While no significant difference was found between normal and DCIS subgroups, the normal/DCIS was statistically significant (P<0.01) from the nodal negative- and nodal positive-invasive breast carcinomas using the ANOVA and subsequent Tukey post-hoc analysis (see Materials and Methods). Further Tukey post-hoc analysis indicated a significant difference (*, P<0.05) between the invasive node negative and invasive node positive breast carcinoma tissues. C. The distribution of staining intensity scores shows the change in staining pattern between normal/DCIS and invasive case types. Scores were grouped as low (0–1), medium (2), and high (3). Data represent the ratio of the number of samples in each group (low, medium, high) to the total number of samples.

Since anoikis resistance is a determinant of tumor progression and metastasis in tumor cells, we tested the possibility that the Bit1 anoikis pathway is suppressed in human mammary cancers. We used immunohistochemistry to examine Bit1 expression in human mammary tumor tissue array consisting of DCIS (Ductal Carcinoma In situ lesions), invasive breast carcinomas, and normal counterpart breast tissues using a rabbit affinity purified anti-Bit1 antibody (Sigma). The specificity of this antibody has been validated by the manufacturer and the Human Protein Atlas Project. The normal mammary epithelium showed moderate to strong cytoplasmic immunoreactivity for Bit1 (Figure 1A i-ii). The majority of DCIS lesions (Figure 1A iii) retained similar Bit1 immunostaining as that of normal breast tissue. Interestingly, in certain cases of DCIS lesions, epithelial cells that had filled the breast duct had completely lost Bit1 expression while those epithelial cells that remain in direct contact with the stroma maintained Bit1 immunoreactivity (Figure 1A iv). In contrast to normal and DCIS breast tissues, invasive breast carcinomas, both nodal negative and nodal positive subgroups, showed reduced cytoplasmic Bit1 immunoreactivity (Figure 1A v–viii). Quantification of the average Bit1 staining (Figure 1B) confirmed the similar Bit1 expression between the normal and DCIS subgroups and the reduction of Bit1 immunoreactivity in invasive nodal negative and positive breast carcinoma tissues as compared to normal/DCIS subgroups. Importantly, we found that the higher grade invasive breast tumor tissues (nodal metastasis positive subgroup) showed a much lower average Bit1 staining (Figure 1B) as compared to invasive node-negative subgroup. Consistent with these findings, analysis of the distribution of staining intensity scores (Figure 1C) showed that while the proportion of staining scores was similar between normal and DCIS subgroups, Bit1 staining frequencies varied dramatically between the normal/DCIS and the invasive node-negative/node-positive subgroups with the node-positive tumor tissues exhibiting the highest frequency of reduced Bit1 immunoreactivity. These findings indicate that Bit1 expression is selectively lost in invasive breast carcinomas, suggesting that loss of Bit1 may accompany the transition from DCIS to invasive carcinoma during the progression of breast cancer.

### Downregulating Bit1 in cultured cells results in enhanced resistance to anoikis

**Figure 2:**
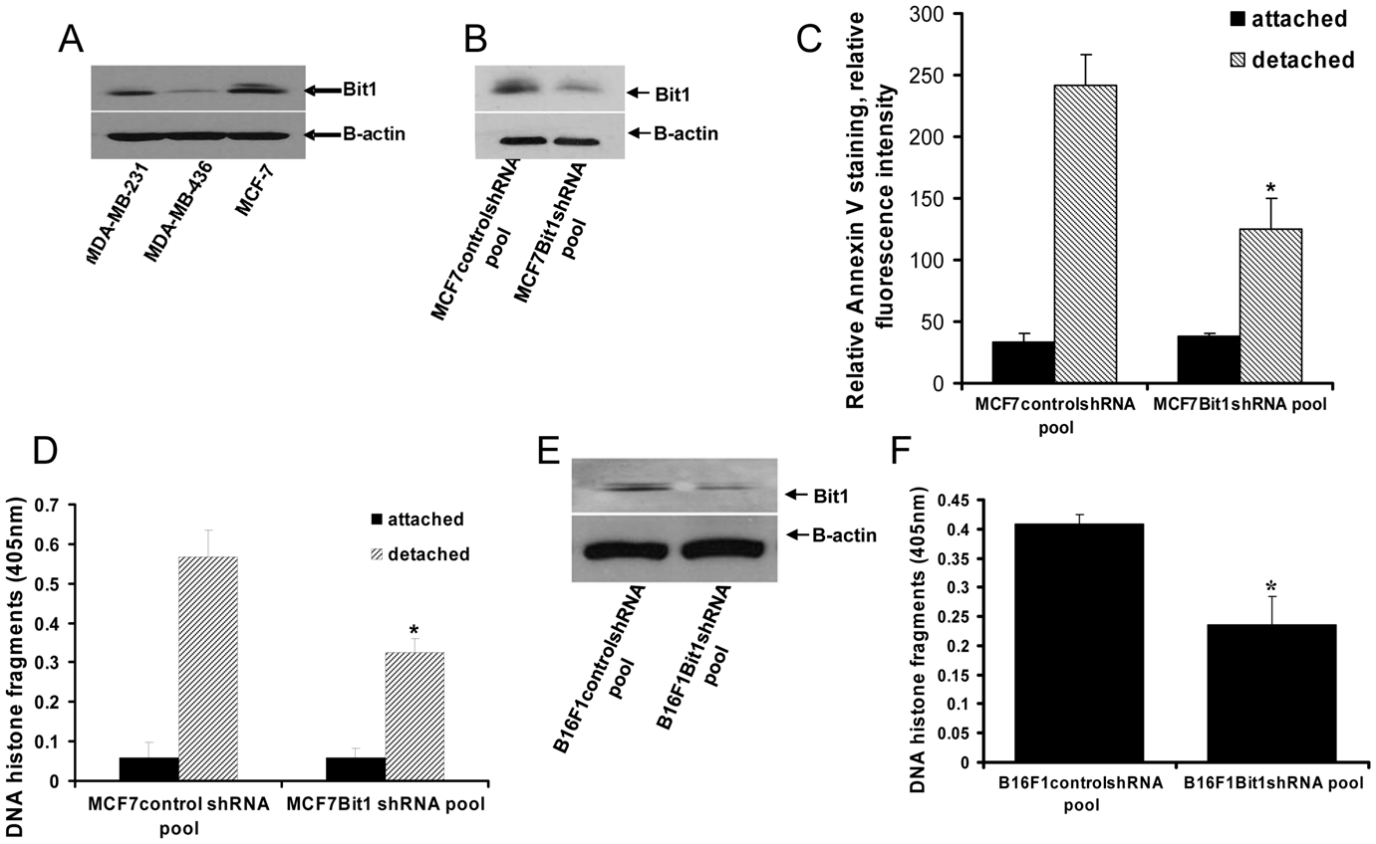
Suppression of Bit1 expression enhances anoikis resistance. A. Total cell lysate derived from each respective breast cancer cell line was subjected to SDS-PAGE and immunoblotting using a specific antibody to Bit1. The membrane was then reprobed with anti-β-actin antibody to confirm equal loading of protein. B. Stable MCF7controlshRNA and Bit1shRNA knockdown pools were generated as described in Materials and Methods, and the total cell lysates derived from controlshRNA and Bit1shRNA knockdown pools were subjected to immunobloting using a specific antibody to Bit1. C and D. MCF7controlshRNA and Bit1shRNA knockdown pools were plated onto a polyhema coated or uncoated tissue culture plates. Following 48 h in culture, cells were the stained with Annexin V, a marker of apoptosis, and the relative fluorescence intensities are shown (C). In D, level of apoptosis was also quantified by measuring the amount of DNA histone fragments (Cell Death Elisa). E and F. The stable control shRNA and Bit1shRNA knockdown pools were also generated from the B16F1 cell line. The resulting B16F1 controlshRNA and Bit1shRNA knockdown pools were subjected to total cell lysate isolation, SDS-PAGE, and immunoblotting against a specific Bit1 antibody to confirm Bit1 downregulation (E). In F, the B16F1 controlshRNA and Bit1shRNA knockdown pools were plated onto a polyhema coated or uncoated tissue culture plates for 48 h, and the level of apoptosis was the quantified by measuring the amount of DNA histone fragments. In C, D, and F, three independent experiments were performed in triplicates. *p<0.05 as compared with control cells (Student's t test).

To address the biological significance of Bit1 downregulation in the aggressive phenotype of breast tumor cells, we suppressed Bit1 expression in the lowly aggressive mammary carcinoma MCF7 cell line which exhibits moderate levels of endogenous Bit1 (Figure 2A). Stable Bit1 knockdown and control clones were generated via infection of MCF7 cells with lentiviral particles containing Bit1 specific- or control- shRNAs. Several control and Bit1 knockdown clones were pooled together to generate the control shRNA and the Bit1 shRNA knockdown pools, respectively. Immunoblotting analysis confirmed the downregulation of endogenous Bit1 expression by 50–70% in the Bit1 knockdown pool (Figure 2B). Since Bit1 is a novel anoikis regulator whose apoptotic function is uniquely regulated by integrin-mediated cell attachment [5], we then examined the impact of Bit1 downregulation on the anoikis sensitivity of MCF7 cells (Figure 2C and 2D). As compared to control pool, the Bit1 knockdown pool exhibited significantly decreased level of apoptosis following culture in suspension for 48 h as evidenced by decreased annexin V staining (Figure 2C) and reduced level of DNA histone fragments (Figure 2D). In stark contrast, no significant differences in the basal apoptosis were observed between the MCF7 Bit1 knockdown and control pools in attached conditions. The observed enhanced anoikis resistance in MCF7-derived Bit1 knockdown pool of cells was also observed in the previously generated stable HeLa cell Bit1 knockdown clones [10] and following stable downregulation of Bit1 expression in the mouse melanoma B16F1 cells (Figure 2E–F).

### Suppressing Bit1 expression changes cell morphology and enhances cell adhesion and migration

**Figure 3:**
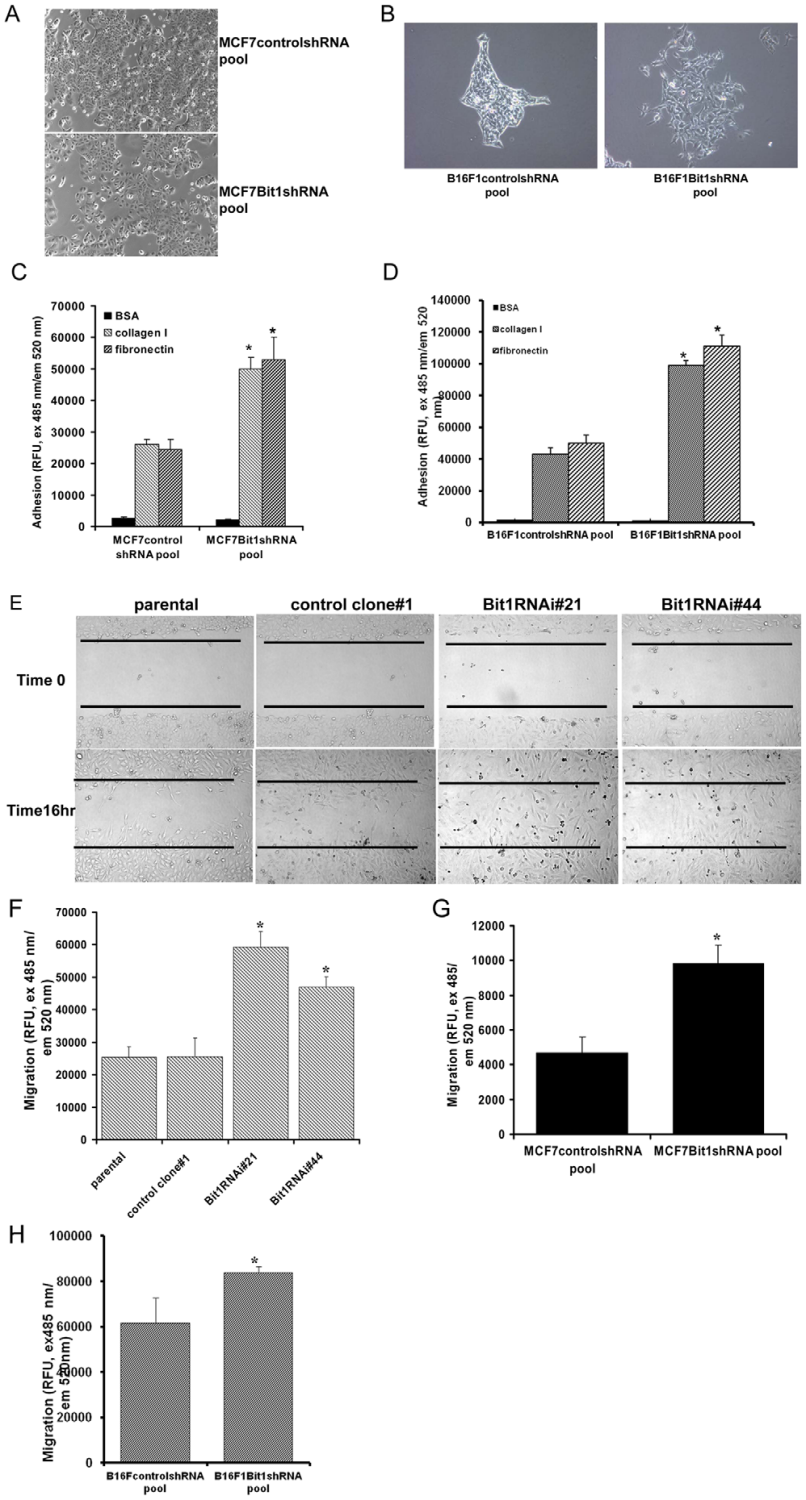
Downregulation of Bit1 results in morphological changes and enhances cellular adhesion and migration. A and B. The morphology of controlshRNA and Bit1shRNA knockdown pools derived from MCF7 (A) and B16F1 (B) was examined by phase contrast microscopy (100× magnification) under normal culture conditions. C and D. Stable control shRNAcontrol and Bit1shRNA knockdown pools derived from MCF7 (C) and B16F1(D) were seeded in 96-well plates precoated with fibronectin, collagen I, or BSA. After 15 min of incubation at 37°C, the number of adherent cells was determined by staining with green fluorescent dye (calcein-AM) followed by fluorescence measurement as described under Materials and Methods (Innocyte Cell Adhesion Assay Kit, EMD Biosciences). E. Stable control and Bit1 Hela knockdown clones were subjected to a wound repair assay. The wound was generated at time 0 h and cell migration into the wound was analyzed by phase contrast microscopy at 16 h. F, G, and H. Control and Bit1 knockdown cells derived from Hela (F), MCF7 (G), and B16F1 (H) were subjected to a QCM 96-well migration boyden chamber assay wherein the number of cells that migrated to the bottom of the insert membrane was quantified by CyQuant Gr dye (Molecular Probes) as described in Materials and Methods. In C, D, F, G, and H, results are representative of three independent experiments, *p<0.05 as compared with the control cells (Student's t test).

We observed that the stable Bit1 knockdown pool derived from both the MCF7 and B16F1 cell lines showed a flatter and stretched morphology relative to the corresponding the control pool (Figure 3A and 3B). Targeted reduction of Bit1 in Hela cells (Figure S1A) also exhibited a fibroblastoid and spindle shape-like morphology. Consistent with their flatter and extended morphology, the stable MCF7 Bit1 knockdown pool (Figure 3C) as well as the B16F1 Bit1 knockdown pool (Figure 3D) adhered more strongly to fibronectin and collagen as compared to the corresponding control cells. A similar increase in cellular adhesive property was also observed in the Hela Bit1 knockdown clones relative to the control clone (Figure S1B). Since adhesion to matrix proteins is also an important step in cell migration [14], [15] we examined the migratory potential of Bit1 knockdown cells. Consistent with their enhanced adhesive properties, the stable Hela knockdown clones showed an increase in migration relative to control clones, as evidenced by *in vitro* wound closure (Figure 3E) and modified Boyden chamber assays (Figure 3F).

It is possible the increased wound closure by the stable Hela Bit1 knockdown clones could be attributed to their increased proliferative ability. To exclude this, we measured the proliferation rates of the different clones *in vitro*. Interestingly, the stable Hela knockdown clones exhibited a slower anchorage-dependent growth rate relative to the control clones (Figure S1C). Such a decrease in growth rate was also observed in MCF7-derived Bit1 knockdown pool as compared to the control pool (Figure S1D). Hence, the observed increase in wound closure by the Hela Bit knockdown clones is attributable to their enhanced migratory ability as confirmed by the modified Boyden chamber migration assay (Figure 3F). To verify that the enhanced migration of Hela cells following Bit1 suppression is not cell line specific, the stable Bit1 knockdown pools derived from MCF7 and B16F1 cells and their corresponding control pool were subjected to modified Boyden Chamber migration assay. Indeed, stable downregulation of endogenous Bit1 in these cells resulted in enhanced migration (Figure 3G for MCF7 and Figure 3H for B16F1 cells) as compared to the control pool.

### Activated Erk contributes to the increased adhesion and migration of Bit1 knockdown cells

**Figure 4:**
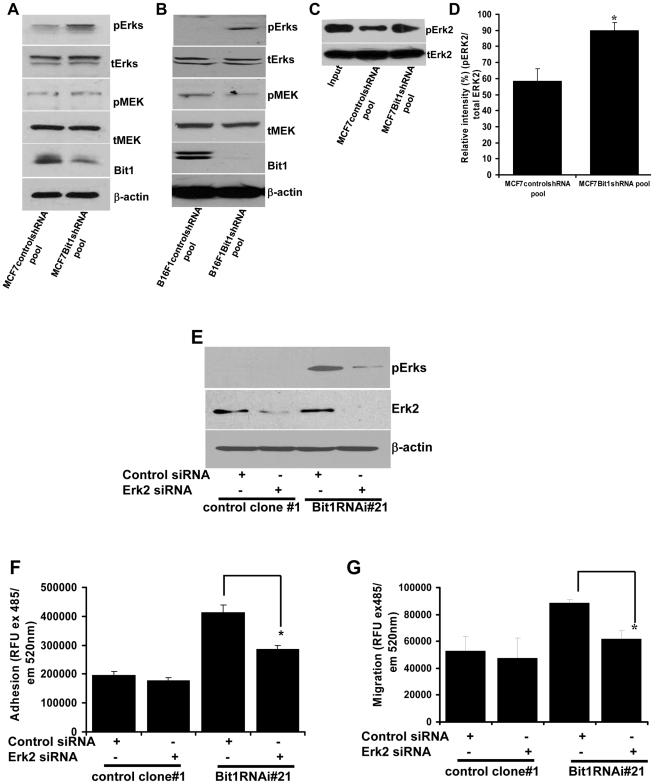
Bit1 negatively regulates Erk activation through decreased Erk phosphatase activity, and Erk down-regulation attenuates the enhanced cell adhesion and motility of Bit1 knockdown cells. A and B. Exponentially growing stable controlshRNA and Bit1shRNA knockdown pools derived from MCF7(A) and B16F1(B) were lysed, and the total lysate was subjected to immunoblotting to detect the phosphorylated Erk (pErk), total Erk(tErk), active Mek (pMek), total Mek (tMek), Bit1, and β-actin. C and D. Total cell lysates from stable MCF7controlshRNA and Bit1shRNA knockdown pools were subjected to an Erk phosphatase assay as described in Materials and Methods. A representative immunoblot of isolated His-6-tagged Erk2 is shown to reveal pErk2 or total Erk2 levels (C). The relative intensity of pErk2/tErk was determined using NIH Image J software, and the values represent the average of at least three independent experiments (D). E, F and G. Stable Hela control clone#1 and Bit1RNAi#21 clone were transfected with control- or Erk2-specific siRNAs; 48 h post-transfection, cells were harvested and subjected to immunoblotting (E) with antibodies against total Erk2 and phosphorylated Erk1/2 (pErks). In parallel, cells were subjected to a fibronectin cell adhesion (F) and QCM boyden chamber migration assays (G) as described in Materials and Methods. In D, F and G, results are representative of three independent experiments, *p<0.05 (Student's t test).

Mouse embryo fibroblasts lacking Bit1 and HeLa cancer cells transiently and stably transfected with Bit1-specific siRNAs exhibited enhanced levels of Erk activation [10]. In particular, the suppression of Bit1 in these cells was associated with a marked increase in the levels of the phosphorylated Erk2 isoform. Consistent with these previous findings, downregulating endogenous Bit1 in MCF7 (Figure 4A) and B16F1 cells (Figure 4B) also resulted in increase of levels of phosphorylated (active) Erk2 with no consistent changes in the levels of active MEK, an upstream regulator of Erk.

To address the mechanism of Erk activation following Bit1 suppression, we examined the Erk-directed phosphatase activity in Bit1 knockdown and control cells using a nonradioactive phosphatase assay with phosphorylated Erk2 as the substrate. Total cell lysate extract from Bit1 knockdown cells exhibited a significant decrease in Erk dephosphorylation as compared with that of control cells (Figure 4C and 4D).

The enhanced Erk activation has been shown to contribute to the anoikis resistance of mouse embryo fibroblasts lacking Bit1 and Bit1 knockdown cells, since siRNA-mediated downregulation of endogenous Erk2 partially restored the anoikis sensitivity of these cells [10]. Here, we examined the effects of inhibiting Erk activation using the Erk2 specific siRNAs on the increased cellular adhesion and migratory potential of Bit1 knockdown cells. The Hela derived Bit1 knockdown cells were chosen for this study due to its high transfectability. Downregulating Erk2 (Figure 4E) significantly attenuated the increased cell adhesion (Figure 4F) and migration (Figure 4G) of the Bit1 knockdown cells with no significant effect on the low adhesion and migratory potential of control cells. These findings indicate that Erk2 activation is likely to contribute to the enhanced adhesion and migration of Bit1 knockdown cells.

### Bit1 downregulation does not significantly influence tumor growth but enhances metastasis

**Figure 5:**
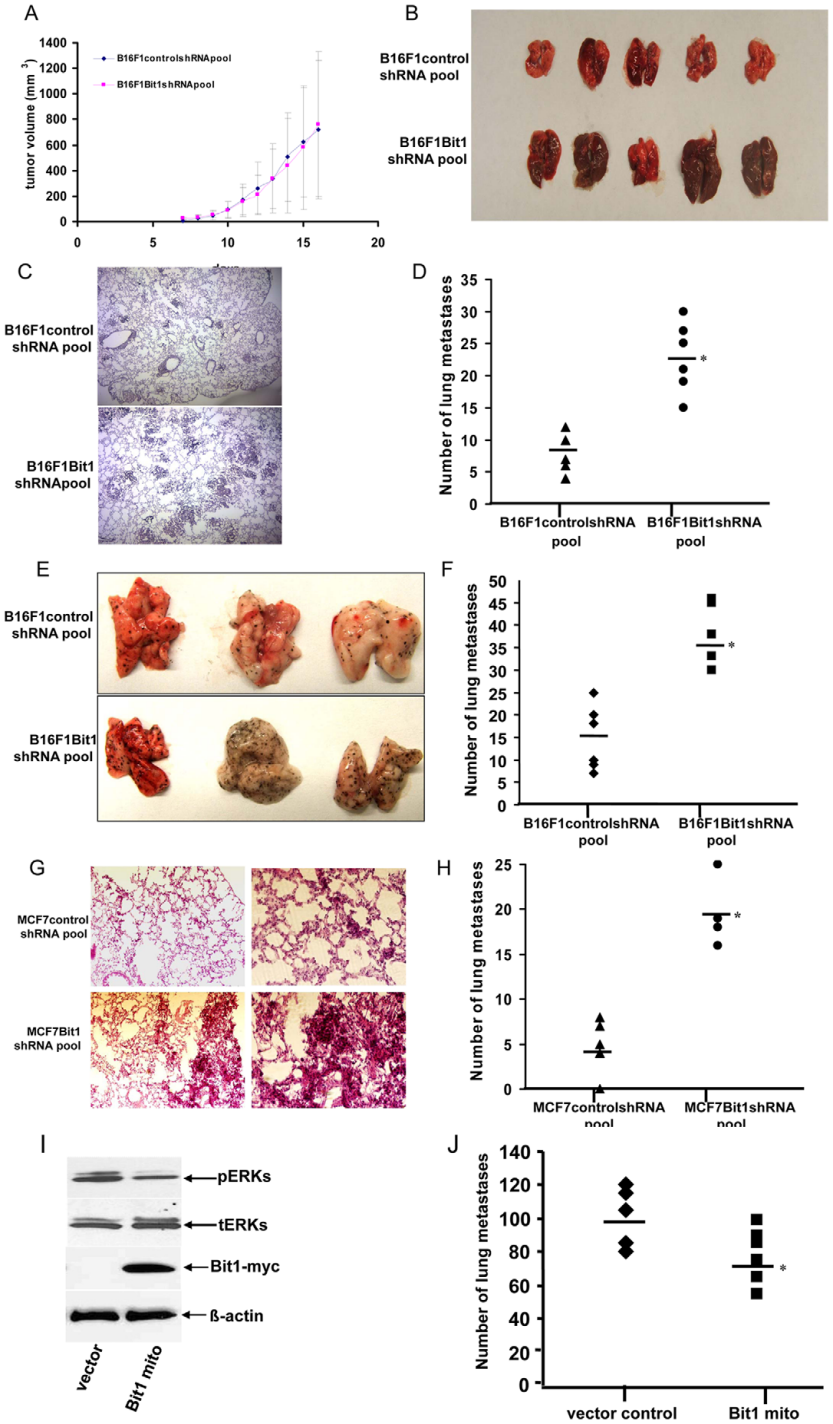
Suppression of Bit1 enhances tumor metastasis with no significant effect on tumor growth *in vivo*. A, B, C, and D. Stable B16F1 controlshRNA and Bit1shRNA knockdown pools were injected subcutaneously in BALB/c nude mice. A total of 20 mice were analyzed with 10 mice injected with control shRNA pool cells while another 10 mice injected with Bit1shRNA pool cells. At the indicated times after injection, tumor volumes were measured (A). At the end of study, the lungs from mice were harvested and photographed (the representative lungs are shown in (B) and random serial sections of paraffin-embedded lung tissue were examined by H&E staining to detect the presence of tumor foci (C) and the numbers of pulmonary metastatic foci were counted (D), *P<0.05 as compared to control shRNA pool. E, F, G and H. Stable control and Bit1 knockdown pools from B16F1 (E and F) and MCF7 (G and H) were injected into the tail vein in BALB/c nude mice. For B16F1, a total of 20 mice were analyzed with 10 tail vein injected with controlshRNA pool and another 10 mice injected with Bit1shRNA. A similar number of mice was used for control shRNA and Bit1shRNA pools derived from MCF7. The mice were sacrificed 30 days post-injection for MCF7cells (20 days for B16F1), and the lungs were harvested and metastatic colonies quantified in serial sections of H&E-stained, paraffin-embedded lung tissue (F and H), *P<0.05 as compared with controlshRNA pool. In E, the representative lungs from mice injected with B16F1 Bit1 knockdown pool showed an obvious increase in metastatic foci on the lung surface relative to that of controlshRNA pool. In G, representative serial sections of H&E-stained, paraffin-embedded lung tissue from mice injected with stable MCF7 controlshRNA or Bit1shRNA knockdown pool are shown. I and J. B16F10 cells were transfected with vector control or C-terminally myc tagged, mitochondrial localized myc-tagged Bit1 construct (Bit1 mito), and 24 h post-transfection, adherent cells were harvested and subjected to immunoblotting against the antibody to myc, pErk, tErk, and β-actin (I). In parallel, vector and mitochondrial Bit1 transfected cells were injected into the tail vein (J) with 10 mice injected with vector control cells and another 10 mice injected with mitochondrial Bit1 transfected cells. 20 days following injection, lungs were harvested and metastatic foci in lung tissue was quantified as described above, *P<0.05 as compared with vector control cells.

The stable MCF7 Bit1 and B16F1 Bit1 knockdown pool produced subcutaneous tumors that grew at the same rate as tumors obtained with the corresponding control cell pools (shown for the B16F1 pool in Figure 5A). Interestingly, the lungs of the mice with tumors from the B16F1 Bit1 knockdown pool showed redness and swelling, suggesting the presence of lung metastases (Figure 5B). Indeed, immunohistochemistry of the lungs derived from these mice showed the presence of numerous microscopic tumor foci while the mice that received the control pool exhibited significantly fewer pulmonary colonies (Figure 5C and D).

To further examine the effect of Bit1 downregulation on metastasis, we subjected the stable B16F1 and MCF7 Bit1 knockdown and the corresponding control pools to experimental tail vein metastasis assays. The lungs of mice that received injections of B16F1 Bit1 knockdown pool (Figure 5E and 5F) and MCF7 Bit1 knockdown pool (Figure 5G and 5H) showed an increase in the number of metastatic foci as compared to the lungs derived from mice injected with control cells. We also observed numerous tumor foci in the lungs of mice injected with HeLa Bit1 knockdown clones, while that of the control clone-treated mice showed no visible signs of tumor infiltrates (Figure S1E). To confirm the effect of Bit1 downregulation on metastatic phenotype, we also restored mitochondrial expression in the highly aggressive and metastatic mouse melanoma B16F10 cell line which shows low levels of endogenous Bit1. Expression of exogenous mitochondrial Bit1 attenuated the high basal Erk activation in B16F10 cells (Figure 5I) and significantly inhibited the ability of these cells to form metastatic foci in lungs following an experimental metastasis assay (Figure 5J). Taken together, these findings implicate a role of Bit1 in metastasis.

### Increased Erk phosphorylation in pulmonary metastases

**Figure 6:**
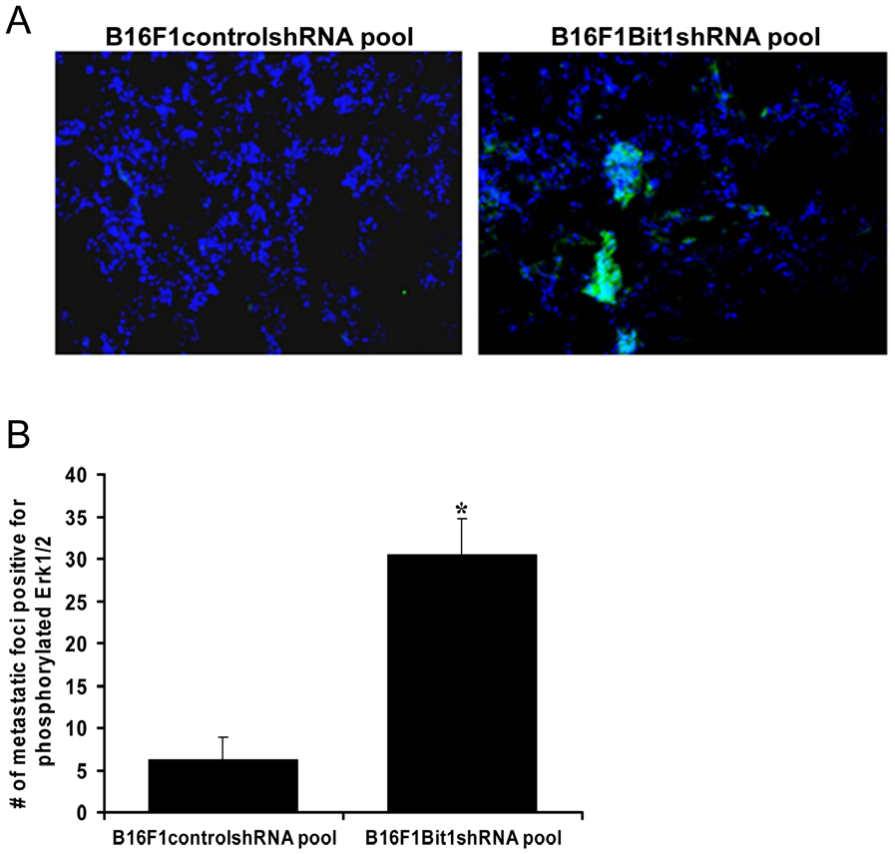
Erk phosphorylation is up-regulated in pulmonary metastatic foci of Bit1 knockdown cells. A. Random serial sections of paraffin-embedded lung tissue derived from mice injected tail vein with stable B16F1controlshRNA or Bit1shRNA knockdown pools were subjected to immunofluorescence analysis of active Erk using anti-phosphorylated Erk antibody (1∶100) as the primary antibody followed by incubation with FITC-conjugated secondary antibody as described in Materials and Methods. Representative staining are shown in (A). B. The number of metastatic tumor foci staining positive for pErk was quantified by examining serial sections of paraffin-embedded lung tissue of mice tail vein injected with stable B16F1controlshRNA or Bit1shRNA knockdown pools. A total of sixty metastatic lesions (30 controlshRNA and 30 Bit1shRNA) from 10 animals (5 tail vein injected with control shRNA pool and 5 tail injected with Bit1shRNA pool) were analyzed, *p<0.01 (Student's t test).

To determine the role of Erk activation in the enhanced metastasis of Bit1 knockdown cells, we stained the lung tissue from mice injected with control and Bit1 knockdown cells using a phospho-specific Erk1/2 antibody. The metastatic foci from Bit1 knockdown cells stained more strongly for phospho-Erk than foci from control cells (Figure 6A). A much higher number of metastatic foci from Bit1 knockdown cells were positive for phospho-Erk 1/2 than foci from control cells (Figure 6B). In primary tumors, the control and Bit1 knockdown cells exhibited similar low levels of phosphorylated Erk staining (data not shown). Thus, the upregulation of Erk phosphorylation in metastatic tumors of Bit1 knockdown cells suggests that Erk activation may be an important effector of metastasis following Bit1 suppression.

## Discussion

Acquisition of anoikis resistance is a determinant of transformation and metastasis in tumor cells. In this paper, we examined the possibility that Bit1 is downregulated in advanced stages of cancer and that suppression or nonfunctionality of the Bit1 anoikis pathway may contribute to tumor progression. Immunohistochemistry of breast tumor tissue arrays revealed that Bit1 is expressed in normal breast epithelial and Ductal Carcinoma In Situ (DCIS) tissues while decrease or loss of Bit1 expression was correlated with advanced invasive carcinoma tissues. *In vitro* studies indicated that downregulating endogenous Bit1 expression enhances the metastasis-associated properties in cultured tumor cells. The Bit1 knockdown cells displayed decreased sensitivity to anoikis, enhanced cell adhesion, increased migration, and high levels of Erk activation. We also showed that suppression of Bit1 conferred enhanced metastatic potential to cancer cells in experimental metastasis assays *in vivo*. These results indicate that Bit1 is a negative regulator of metastasis, likely through reduced Erk activation.

Our findings here illustrating Bit1 as a metastasis suppressor are consistent with and reminiscent of a recent publication demonstrating that AES, a pro-apoptotic binding partner of Bit1, is a suppressor of colon carcinoma metastasis and has no effect on tumorigenicity [13]. Interestingly, this paper also showed that metastasis suppression by AES is through inhibition of tumor cell migration, which is one of the pro-metastatic phenotypes observed in Bit1 knockdown cells. Considering that AES is a critical regulator of the Bit1 pathway, a possibility remains that AES acts in conjunction with Bit1 (or vice versa) to suppress tumor metastasis.

Tumor metastasis is a multistep process involving migration and invasion of the extracellular matrix (ECM), adhesive interactions with ECM components, intravasation through the vessel wall, circulating in the blood vessels, and ultimately lodging into secondary sites [16]. Importantly, while in transit through lymphatic and blood circulation, metastatic cells must survive in the absence of cell attachment-induced survival signals. Here, we show that tumor cells with knocked down Bit1 exhibit several key properties underlying the metastatic process. First, the Bit1 knockdown cells were resistant to anoikis, which may contribute to the survival of these cells in the absence of cell attachment during metastasis. Downregulating Bit1 also increased cell attachment to the matrix proteins fibronectin and collagen. Although tumor cell adhesion plays a complex role in metastasis [17], the enhanced adhesive properties coupled to the enhanced survival capacity may allow the Bit1 knockdown cells to adhere to the target tissue during metastasis. The Bit1 apoptotic function is uniquely controlled by an integrin mediated signalling pathway [5]. The increased cellular adhesion following Bit1 suppression suggests that Bit1 may regulate integrin activity through a feedback mechanism. In agreement with this possibility, activation of MAPK/Erk pathway, which Bit1 counteracts, activates integrin [18], [19].

Another important metastasis-associated property exhibited by Bit1 knockdown cells is their increased motility. Although our data indicate that the increase in motility is in part dependent on Erk activation, this increase in motility may be a consequence of the enhanced adhesive properties because tumor cell adhesion to extracellular matrix components is important in cell migration [17]. Active migration of tumor cells is a prerequisite of metastasis and is one of the critical factors in epithelial- mesenchymal transition (EMT), a process associated with metastatic progression [20]. The morphological changes observed in Bit1 knockdown HeLa cells are consistent with the EMT phenotype, but it remains to be investigated whether Bit1 affects the EMT process. Our recent findings of the loss of E-cadherin and upregulation of N-cadherin expression (Figure S1F) in Hela Bit1 knockdown cells support this possibility.

Interestingly, the Bit1 knockdown cells showed decreased cell growth in adherent culture conditions as compared to control cells (Figure S1C–S1D [21]). The reduction of growth was observed in several cancer cell lines following suppression of endogenous Bit1 protein, suggesting that this effect is specific to Bit1 downregulation. Based on crystal structural studies [22], Bit1 contains a peptidyl tRNA hydrolase domain which is distinct from its apoptotic domain. It is conceivable that the native mitochondrial Bit1 may impact cellular translation process and subsequent cell growth via its tRNA hydrolase enzymatic function. Consistent with this notion, ectopic overpression of the tRNA hydrolase domain of Bit1 in cultured cells resulted in enhanced cell growth (unpublished data). This dual functionality of Bit1 is reminiscent of another mitochondrial protein namely Apoptosis Inducing Factor (AIF) in controlling cellular life and death. AIF exhibits pro-apoptotic caspase-independent activity following its release from the mitochondria and functions as a survival factor via its oxidoreductase activity when localized in native mitochondria [23].

The metastasis-associated properties of the Bit1 knockdown cells appear to involve the Erk survival pathway. Bit1 is a negative regulator of Erk activity; mouse embryonic fibroblasts from Bit1 knockout mice, as well as cultured HeLa cells treated with Bit1-specific siRNAs, exhibit enhanced levels of active (phosphorylated) Erk [10]. This Erk activation contributes in part to the enhanced anoikis resistance of the cells. In this report, downregulation of Bit1 in MCF7 and B16F1 cells also increased the levels of phosphorylated Erk2, the isoform which is specifically affected by Bit1 [10]. Furthermore, suppressing Erk activity via Erk2 specific siRNAs attenuated the enhanced adhesion and migratory capacity of Bit1 knockdown cells, indicating the involvement of the Erk pathway in Bit1 regulation of cell adhesion and motility. Based on the recent report demonstrating that the anti-invasive and anti-metastasis function of AES is through inhibition of the Notch signalling [13], it remains to be determined whether Bit1 also regulates this pathway.

The mechanism of Erk regulation by Bit1 remains to be fully elucidated, but available evidence suggests that Bit1 inhibits Erk activation through induction of Erk-specific phosphatases. The Bit1 knockout and knockdown cells with elevated Erk activation show no changes in activation of the upstream regulators of Erk, the AKT and MEK kinases, but Erk-directed phosphatase activity is significantly decreased in these cells (Figure 4C-4D [10]). Interestingly, cell adhesion, which regulates the apoptotic activity of Bit1, also regulates Erk phosphatase function [24], [25]. In particular, cell adhesion decreases, and detachment increases Erk-directed phosphatase activity. To date, several phosphatases for Erk have been identified, including CDC25A, He-PTP, and several MAP kinase phosphatases (MKPs) [24], [25]. It is noteworthy that the activity of MKP3/DUSP6 has been shown to be regulated by cell adhesion and suppresses the anchorage-independent activation of Erk2 induced by vinexin β, a focal adhesion scaffold protein [25], making this phosphatase a likely Bit1 target. Interestingly, the Scansite 2.0 Motif program [26] identifies an Erk binding site in Bit1. Whether Bit1 associates with Erk and how such an interaction impinges on Erk activity remain to be investigated.

Consistent with the *in vitro* findings, the Bit1 knockdown cells exhibited enhanced capacity to produce metastatic tumors as compared to control cells. In particular, we observed increased metastasis both from subcutaneous tumors and from intravenously injected Bit knockdown tumor cells. In keeping with the *in vitro* results suggesting Erk involvement in the Bit1 effects, the metastatic tumors derived from Bit1 knockdown cells exhibited significantly positive staining for phosphorylated Erk. A number of reports documents the importance and requirement of the Erk pathway in promoting cancer cell motility, invasion, and metastasis in various tumors [7], [8], [9]. In particular, analyses of the Erk activation in the experimental metastasis model of MEK transformed cells demonstrated a requirement for Erk activity in the acquisition of advanced stages of cancer progression, particularly in promoting tumor cell invasion and metastasis [9], [27]. Given that Erk activity underlies in part the observed enhanced anoikis resistance, cellular adhesion and motility in Bit1 knockdown cells *in vitro*, together with the enhanced Erk phosphorylation in pulmonary tumors *in vivo*, reduction of Bit1 expression may enhance the metastatic ability of cells at least in part by potentiating Erk signalling.

Interestingly, we found no significant differences in the basal Erk activation in primary tumors derived from control and Bit1 knockdown cells. This finding is consistent with the possibility that the effect of Bit1 on Erk activity is dispensable during primary tumor growth. The complex rich microenvironment and the presence of multiple redundant oncogenic survival pathways at the primary tumor site *in vivo* may reduce the requirement for and hence, mask the effect of Bit1 on Erk-mediated signaling during tumor growth. In line with this notion, non-invasive, benign like clonal expansion or growth of MEK transformed cells did not require Erk activation and was associated with diminished levels of Erk [9], [27]. Given the critical role of the Erk activity in invasion and metastatic processes [9], [27], we propose that the effect of Bit1 knockdown on Erk signalling becomes potentiated or more evident when cells loose contact with the primary site during invasion and metastasis. Endowed with enhanced Erk activation potential, the Bit1 knockdown cells may then be selected for increased metastatic potential. In support of this hypothesis, we found that downregulation of Bit1 specifically enhanced the metastatic property of tumor cells with no significant impact on their tumorigenicity, and the metastatic foci of Bit1 knockdown cells showed increased Erk activation.

In summary, we have uncovered that downregulation of Bit1 expression conferred tumor cells with pro-metastatic phenotypes *in vitro* and specifically potentiated metastasis *in vivo*. Thus, the suppression of Bit1 in human tumor specimens, as evidenced in advanced stages of human breast cancer (Figure 1A–1C) may contribute to a more aggressive and metastatic disease.

## Materials and Methods

### Cell culture and transfection assays

MCF7, B16F1 and B16F10 cell lines from American Type Culture Collection (ATCC) were cultured in Dulbecco’s modified Eagle’s medium (DMEM) with glutamine containing 10% fetal bovine serum, penicillin, and streptomycin, while the stable Hela control and Bit1 knockdown clones were cultured in DMEM glutamine containing 10% fetal bovine serum, penicillin, and streptomycin with 250 ug/ml G418 [10]. Transfections were carried out with lipofectamine 2000 (Invitrogen) in OPTI-MEM (Invitrogen) according to the manufacturer's protocol with cells plated 18 hr before transfection. The total amount of plasmid used per transfection was normalized with the corresponding empty vector constructs.

### Chemical reagents, antibodies, and plasmids

Poly(2-hydroxyethyl methacrylate (Polyhema) and the mouse monoclonal anti-β-actin antibody were obtained from Sigma Chemical Co. (St. Louis, Mo.). The E-cadherin, N-cadherin, anti-total Erk, anti-phosphoErk, anti-Erk2, anti-MEK, and anti-phospho MEK polyclonal antibodies were obtained from Cell Signaling Technology (Beverly, MA). FITC-conjugated secondary anti-rabbit antibody was purchased from Molecular Probes (Eugene, OR). The anti-Bit1 antibody and the mammalian expression vector encoding for mitochondrial Bit1 were generated as described previously [5]. The specific antibody to Bit1 used for immunoblotting was generated as described previously [5], and the affinity purified rabbit anti-Bit1 antibody (HPA012897, Sigma) was used for immunohistochemisry studies.

### Lentiviral shRNA infection

MCF7 and B16F1 cells were infected with control- or Bit1-shRNA lentiviral particles (Santa Cruz Biotechnology) in a six-well plate format in the presence of polybrene (5 ug/ml). 24 h post-infection, cells were treated with puromycin (2 ug/ml and 4 ug/ml for MCF7 and B16F1, respectively) to select for stable control and Bit1 knockdown clones. Several puromycin resistant control and Bit1 knockdown clones were harvested by ring-cloning, and the level of Bit1 knockdown was confirmed by immunoblotting against a specific Bit1 antibody. Two controlshRNA clones and three positive Bit1shRNA knockdown clones were pooled together to generate the controlshRNA pool and Bit1shRNA pool, respectively. The resulting controlshRNA and Bit1shRNA pools were subjected to immunoblotting using a specific antibody to Bit1 to confirm the downregulation of Bit1 expression.

### SiRNA transfection

The control and ERK2 specific siRNAs were purchased from Santa Cruz Biotechnology. To downregulate Erk2 in a stable Bit1 Hela knockdown clone, 2×10^5^ cells were transfected with 25 µM of ERK2 siRNAs with the use of Lipofectamine 2000 reagent. 48 hrs post-transfection, cells were harvested and analyzed by i) immunoblotting with antibodies against Erk2 (Cell Signaling) to confirm the downregulation of ERK2 or ii) migration assay as described below.

### Analysis of anoikis

To assess for anoikis cell death, cells were plated in Poly-HEMA coated 96 well plates in complete growth medium containing 0.5% methylcellulose at a density of 1.0×10^4^/well for 48 h. Suspension cultures were prepared by plating cells on dishes coated with poly-HEMA (Sigma) and culturing in serum-containing medium Cells were collected and subjected to an apoptosis assay [6]. Apoptosis was assessed by determining the level of cytosolic nucleosomal fragments with the use of Cell Death Detection ELISA Kit according to the manufacturer's instructions (Roche Molecular Biochemicals) and by Annexin V staining (Biovision) which was analyzed by flow cytometry (BD FACS StarPlus) as prescribed by the manufacturer.

### Cell proliferation, cell adhesion and migration assays

To determine cell proliferation, cells were plates in a volume of 150 µl at a density of 2,000 cells per well in 96-well plates. At each indicated time, the number of metabolically active cells was measured with the use of MTT (Thiazolyl Blue Tetrazolium Bromide, Sigma). Briefly, fifteen µl of MTT solution (5 mg/ml in PBS) was added and further incubated for 4 h. The resulting MTT formazan was solubilized by addition of 100 µl of SDS solution (20% in 10 mM HCL), and the absorbance was measured 24 h at 550 nm and a reference wavelength of 690 nm using a microplate reader. In cell adhesion experiments, 2×10^4^ cells were seeded in 96-well plates precoated with fibronectin, collagen type 1, or BSA (Innocyte ECM Cell Adhesion Assay Kit, EMD Biosciences) for 15 min at 37C, and cells were subsequently twice washed with PBS to remove non-adherent cells. Following extensive washing, attached cells were quantified by staining with the green fluorescent dye calcein-AM according to manufacturer's protocol. The migration potential cells were determined by a wound closure and a QCM Chemotaxis 96-well cell migration assays. In wound closure experiments, cell monolayers were scarred with a sterile micropipette tip and incubated for another 16 h. For each sample, three defined areas were monitored during the time period and photographs were taken at time 0 h and 16 h (magnification, X100). Using the QCM 96-well migration assay kit (Chemicon), 2.5×10^4^ cells were added into the top chamber of a boyden chamber in 100 ul of serum free DMEM medium. After 24 h of incubation, migratory cells on the bottom of the insert were dissociated from the membrane using the Cell Detachment Buffer according to manufacturer's protocol. The cells are then lysed and detected by fluorescent CyQuant GR dye (Molecular Probes).

### Protein preparation and western blotting assays

Protein preparation and western blotting were performed as described previously [6]. Briefly, cells were harvested 24–48 hr after transfection with various constructs or siRNAs by adding ice-cold NP-40 lysis buffer (1% NP-40; 20 mM Tris-HCL [pH 7.4]; 150 mM NaCl; 10% glycerol, 2 mM sodium vanadate; 1 mM henylmethylsulfonyl fluoride; 10 µg/ml leupeptine; and 5 µg/ml aprotinin) and incubating at 4°C for 20 min. For immunoblot analysis, equal amounts of proteins were resolved on 4–20% gradient Tris-glycine gels (Invitrogen) and electrophoretically transferred to nitrocellulose membrane. The membranes were incubated with primary antibodies overnight at 4°C followed by secondary antibodies conjugated with horseradish peroxidase. Membranes were developed using the ECL detection system.

### Erk Phosphatase Assay

The *in vitro* Erk phosphatase assay is based on detecting dephosphorylation of a purified, phosphorylated, His_6_-tagged Erk2 upon incubation with total cell lysate [10]. Briefly, 100 µg of cell lysate prepared without phosphatase inhibitors was diluted 1∶4 in phosphatase assay buffer [10 mM MgCl_2_, 10 mM Hepes (pH 7.4), and 10 µM Mek inhibitor UO126] and incubated with 30 ng of recombinant phosphorylated His_6_-tagged Erk2 (Biomol) at room temperature for 30 min. The reaction was terminated by adding 8 M urea (pH 8.6) containing 10 mM imidazole, and the samples were placed on ice. The His-Erk2 was subsequently captured by adding nickel-conjugated agarose and incubating at 4°C for 90 min. The samples were washed three times with 8 M urea (pH 8.6) and 10 mM imidazole and two times in 300 mM NaCl_2_ and 25 mM Tris (pH 7.5). The amount of remaining phosphorylated His-Erk2 was determined by immunoblotting using a specific antibody to phosphorylated ERK. The amount of total ERK was also determined using a antibody against total ERK to account for protein loading.

### *In vivo* tumorigenesis and metastasis assays

All procedures were done according to protocols approved by the Institutional Committee for Use and Care of Laboratory Animals of University of California, Santa Barbara (UCSB Approved Protocol 5-010-733 R). Eight-week-old female athymic nude mice (BALB/c) were used for the tumorigenesis assays. The indicated MCF7, or B16F1 cells (1×10^6^) were injected subcutaneously into the flank. The tumor sizes were measured periodically with a calliper, and tumor volume was determined with the formula (d1×d2^2^)/2 where d1 represents the larger diameter and d2 the smaller diameter. Mice were sacrificed when the primary tumors reached 2 cm in diameter and tumors and lungs were excised and subjected to immunohistochemistry for analysis of metastasis. In experimental metastasis assay, 1×10^6^ cells (for MCF7 and HeLa) or 0.5×10^6^ cells (for B16F series) in 100 µl PBS were injected into the lateral vein of athymic BALB/c nude mice. Thirty days (for MCF7 and Hela cells) and twenty days (for B16F series cells) after the injection, the mice were euthanized. The lungs were dissected out, photographed, fixed in 4% PFA overnight, cryoprotected in 30% sucrose in PBS and frozen in OCT embedding media (Tissue Tek). Serial sections of the lungs were stained with H&E, and the number of pulmonary colonies was counted under a light microscope.

### Immunofluorescence microscopy

For immunostaining, 5 µm lung tumor tissue section slides were deparafinized in xylene for 10 minutes, hydraded through graded alcohols, rinsed with 1xPBS, blocked in 5% serum/2% albumin in PBS for 1 h at room temperature, and then followed by incubation with the primary antibody diluted in blocking buffer for overnight at 4°C. The primary antibodies were subsequently detected by incubation with secondary antibodies coupled to FITC for 1 h at room temperature. Nuclei were visualized by counterstaining with 4′6-diamidino-2-phenylindole (DAPI). Samples were then analyzed using a Carl Zeiss fluorescence microscope.

### Human breast tumor array analysis

Breast tumor tissue array slides containing intraductal carcinomas, invasive ductal carcinoma (lymph node negative), and metastatic invasive ductal carcinoma to the lymph node (lymph node positive) with matched normal breast epithelial tissue were obtained from US Biomax, Inc. (Rockville, MD). Based on two tissue microarray slides, a total of 55 intraductal carcinomas, 45 lymph node negative invasive ductal carcinomas, and 40 lymph node positive invasive ductal carcinomas were included for analysis. The immunohistochemistry procedure was performed by Biomax Inc. Briefly, tissue array slides were deparaffinised, hydrated and subjected to antigen retrieval. The slides were then incubated in 2.5% normal horse serum for 30 min at room temperature followed by incubation with the purified rabbit anti-Bit1 antibody (1∶100 dilution) for 1 h at room temperature. Rabbit normal serum was used as negative control antibody to replace the primary antibody on control slide with 1 hr incubation. Tissue array slides were then washed and incubated with ImmPRESS reagent (Vector Laboratories) followed by treatment with peroxidise substrate DAB solution (DAKO Cytomation). Each of the experimental and control slide was scored for average staining intensity by two investigators with no knowledge of the pathologic status of the samples. These investigators scored staining intensity as 0, no staining; 1 low staining; 2 medium staining; or 3 high staining.

### Statistical Analysis

Data are presented as means (±S.E.). For western blots and anoikis assays, experiments were performed at least three times with duplicates. Statistical differences between groups were established at a P value<0.05 using the two-tailed Student's t test. For breast tumor tissue array analysis, a one-way ANOVA with subsequent post hoc testing using the Tukey-Kramer multiple comparison test was used to compare the average staining intensity of each case type. ANOVA analysis indicated a significant difference (P<0.01) between the normal/DCIS and the node negative and node positive invasive subgroups. Additional post-hoc analysis indicated no significant difference between normal and DCIS subgroups (P>0.1), but significant differences between normal/DCIS and invasive node negative (P<0.01) and invasive node positive (P<0.01) subgroups. Further post-hoc testing showed significant difference (P<0.05) between the invasive node negative and invasive node positive subgroups. All calculations were done using the NCSS statistical software (NCSS, Kasville, UT).

## Supporting Information

Figure S1 **Effects of stable suppression of Bit1 in Hela and MCF7 cells.** A. The morphology of exponentially growing stable Bit1 knockdown and control clones previously established in the Hela cancer cell line [10] was examined by phase contrast microscopy (100× magnification). B. The Hela control and Bit1 knockdown clones were seeded in 96-well plates precoated with fibronectin, collagen I, or BSA. After 15 min of incubation at 37°C, the number of adherent cells was determined by staining with green fluorescent dye, calcein-AM followed by fluorescense measurement as described under Materials and Methods. C and D. Stable Bit1 knockdown and control cells derived from Hela (C) and MCF7(D) parental lines were subjected to MTT assay (see Materials and Methods) to quantify their anchorage-dependent growth. E. The stable HeLa Bit1 knockdown and control clones were injected into the tail vein using 10 mice per clone, and 30 days after injection the lungs were harvested and metastatic colonies were quantified in random serial sections of H&E-stained, paraffin-embedded lung tissue. F. Exponentially growing stable HeLa Bit1 knockdown and control clones were lysed, and the resulting total lysate was subjected to immunoblotting using the antibodies against E-cadherin, N-cadherin, and β-actin. In B, C, D and E, results are representative of three independent experiments, *p<0.05 (compared to control cells, Student's t test).
(TIF)
