# Supplementary figures and images for: Metastasis of Tumor Cells Is Enhanced by Downregulation of Bit1
Source: PLoS One. 2011 Aug 23;6(8):e23840. doi: 10.1371/journal.pone.0023840 (PMC3160313; doi:10.1371/journal.pone.0023840)

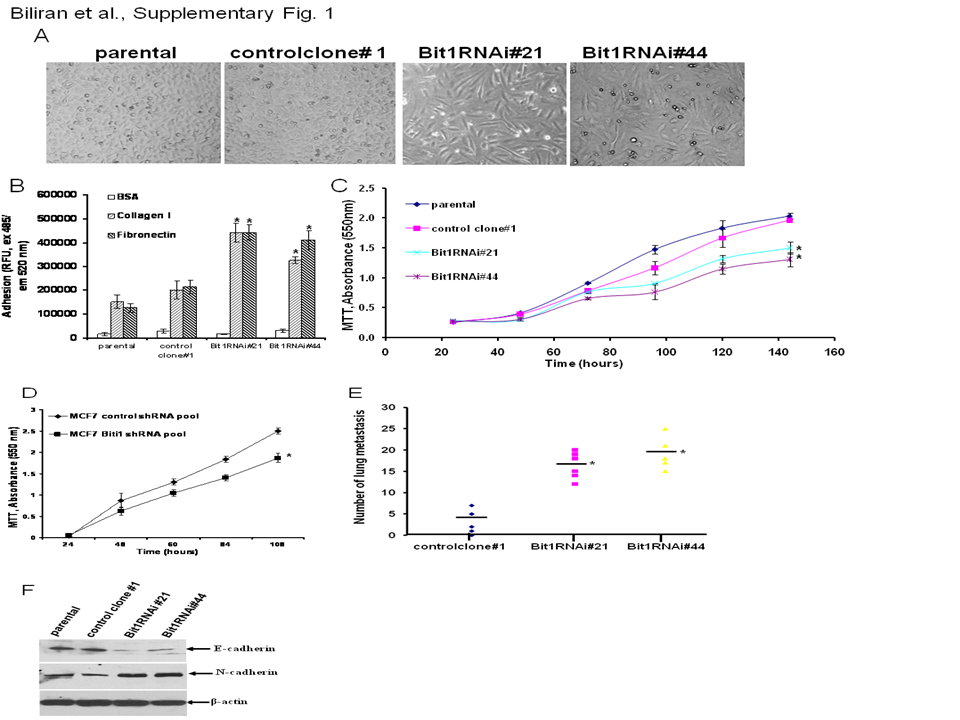

Supplement: Figure S1 — Effects of stable suppression of Bit1 in Hela and MCF7 cells. A. The morphology of exponentially growing stable Bit1 knockdown and control clones previously established in the Hela cancer cell line [10] was examined by phase contrast microscopy (100× magnification). B. The Hela control and Bit1 knockdown clones were seeded in 96-well plates precoated with fibronectin, collagen I, or BSA. After 15 min of incubation at 37°C, the number of adherent cells was determined by staining with green fluorescent dye, calcein-AM followed by fluorescense measurement as described under Materials and Methods. C and D. Stable Bit1 knockdown and control cells derived from Hela (C) and MCF7(D) parental lines were subjected to MTT assay (see Materials and Methods) to quantify their anchorage-dependent growth. E. The stable HeLa Bit1 knockdown and control clones were injected into the tail vein using 10 mice per clone, and 30 days after injection the lungs were harvested and metastatic colonies were quantified in random serial sections of H&E-stained, paraffin-embedded lung tissue. F. Exponentially growing stable HeLa Bit1 knockdown and control clones were lysed, and the resulting total lysate was subjected to immunoblotting using the antibodies against E-cadherin, N-cadherin, and β-actin. In B, C, D and E, results are representative of three independent experiments, *p<0.05 (compared to control cells, Student's t test). (TIF) [file pone.0023840.s001.tif]
